# Biodistribution and pharmacokinetics of^111^In-DTPA-labelled pegylated liposomes in a human tumour xenograft model: implications for novel targeting strategies

**DOI:** 10.1054/bjoc.1999.1232

**Published:** 2000-06-15

**Authors:** K J Harrington, G Rowlinson-Busza, K N Syrigos, P S Uster, R M Abra, J S W Stewart

**Affiliations:** ICRF Oncology Unit, Imperial College of Science, Technology and Medicine, Hammersmith Hospital, 150 DuCane Road, London, W12 0HS, UK; Molecular Medicine Program, Guggenheim 1836, Mayo Clinic, 200 1st Street SW, Rochester, Minnesota, 55902, USA; SEQUUS Pharmaceuticals Inc., Menlo Park, CA, USA; Department of Radiotherapy, Charing Cross Hospital, Fulham Palace Road, London, W6, UK

**Keywords:** biodistribution, head and neck cancer, pegylated liposomes, pharmacokinetics, tumour targeting, xenograft tumour

## Abstract

The biodistribution and pharmacokinetics of^111^In-DTPA-labelled pegylated liposomes in tumour-bearing nude mice was studied to examine possible applications of pegylated liposome-targeted anti-cancer therapies. Nude mice received an intravenous injection of 100 μl of^111^In-DTPA-labelled pegylated liposomes, containing 0.37–0.74 MBq of activity. The t _1/2α_ and t _1/2β_ of^111^In-DTPA-labelled pegylated liposomes were 1.1 and 10.3 h, respectively. Tumour uptake was maximal at 24 h at 5.5 ± 3.0% ID g^–1^. Significant reticuloendothelial system uptake was demonstrated with 19.3 ± 2.8 and 18.8 ± 4.2% ID g^–1^at 24 h in the liver and spleen, respectively. Other sites of appreciable deposition were the kidney, skin, female reproductive tract and to a lesser extent the gastrointestinal tract. There was no indication of cumulative deposition of pegylated liposomes in the lung, central nervous system, musculoskeletal system, heart or adrenal glands. In contrast, the t _1/2α_ and t _1/2β_ of unencapsulated^111^In-DTPA were 5 min and 1.1 h, respectively, with no evidence of accumulation in tumour or normal tissues. Incubation of^111^In-DTPA-labelled pegylated liposomes in human serum for up to 10 days confirmed that they are very stable, with only minor leakage of their contents. The potential applications of pegylated liposomes in the arena of targeted therapy of solid cancers are discussed. © 2000 Cancer Research Campaign


*British Journal of Cancer* (2000) **83**(2), 232–238
© 2000 Cancer Research Campaign
doi: 10.1054/ bjoc.1999.1232, available online at http://www.idealibrary.com on IDEAL®

### Biodistribution and pharmacokinetics of $^{111}$In-DTPA-labelled pegylated liposomes in a human tumour xenograft model: implications for novel targeting strategies

**KJ Harrington[1,2], G Rowlinson-Busza[1], KN Syrigos[1], PS Uster[3], RM Abra[3] and JSW Stewart[4]**

[1]ICRF Oncology Unit, Imperial College of Science, Technology and Medicine, Hammersmith Hospital, 150 DuCane Road, London W12 0HS, UK; [2]Molecular Medicine Program, Guggenheim 1836, Mayo Clinic, 200 1st Street SW, Rochester, Minnesota 55902, USA; [3]SEQUUS Pharmaceuticals Inc., Menlo Park, CA, USA; [4]Department of Radiotherapy, Charing Cross Hospital, Fulham Palace Road, London W6, UK

**Summary** The biodistribution and pharmacokinetics of $^{111}$In-DTPA-labelled pegylated liposomes in tumour-bearing nude mice was studied to examine possible applications of pegylated liposome-targeted anti-cancer therapies. Nude mice received an intravenous injection of 100 μl of $^{111}$In-DTPA-labelled pegylated liposomes, containing 0.37–0.74 MBq of activity. The $t_{1/2\alpha}$ and $t_{1/2\beta}$ of $^{111}$In-DTPA-labelled pegylated liposomes were 1.1 and 10.3 h, respectively. Tumour uptake was maximal at 24 h at 5.5 ± 3.0% ID $g^{-1}$. Significant reticuloendothelial system uptake was demonstrated with 19.3 ± 2.8 and 18.8 ± 4.2% ID $g^{-1}$ at 24 h in the liver and spleen, respectively. Other sites of appreciable deposition were the kidney, skin, female reproductive tract and to a lesser extent the gastrointestinal tract. There was no indication of cumulative deposition of pegylated liposomes in the lung, central nervous system, musculoskeletal system, heart or adrenal glands. In contrast, the $t_{1/2\alpha}$ and $t_{1/2\beta}$ of unencapsulated $^{111}$In-DTPA were 5 min and 1.1 h, respectively, with no evidence of accumulation in tumour or normal tissues. Incubation of $^{111}$In-DTPA-labelled pegylated liposomes in human serum for up to 10 days confirmed that they are very stable, with only minor leakage of their contents. The potential applications of pegylated liposomes in the arena of targeted therapy of solid cancers are discussed. © 2000 Cancer Research Campaign

**Keywords**: biodistribution; head and neck cancer; pegylated liposomes; pharmacokinetics; tumour targeting; xenograft tumour

Liposomes are self-assembling colloidal particles comprising a lipid bilayer and an enclosed fraction of the surrounding aqueous medium (Lasic and Papahadjopoulos, 1995). After their original description more than 30 years ago (Bangham et al, 1965), it was postulated that they would become an important vehicle for targeted drug delivery in a range of clinical situations including cancer therapy (Gregoriades et al, 1974). However, subsequent studies highlighted the limitations of these conventional liposomes, in particular their rapid clearance by the reticulo-endothelial system (RES), unpredictable patterns of extravasation and lack of long-term physicochemical stability (Gabizon 1994). As a result, clinical applications of liposomes have been largely restricted to situations in which targeting of the RES is desirable, such as the treatment of systemic protozoal and fungal infections (Russo et al, 1996; Ng and Denning, 1995).

However, in the last decade, the development of sterically stabilized liposomes has renewed interest in the use of liposomes to treat cancer. The lipid bilayer of sterically stabilized liposomes is modified by the addition of glycolipids (Allen and Chonn, 1987), phosphatidylinositol (Gabizon and Papahadjopoulos, 1988) or methoxypoly(ethylene glycol) (MPEG)-derivatized lipid (Papahadjopoulos et al, 1991). The latter furnishes a steric barrier against interactions with plasma proteins, such as opsonins and lipoproteins, and cell-surface receptors. As a result, pegylated liposomes are better able to evade the RES and remain in circulation for prolonged periods, thereby conferring on entrapped agents the pharmacokinetic profile of the lipid carrier, rather than that of the free drug (Gabizon and Papahadjopoulos, 1988; Paphadjopoulos et al, 1991; Gabizon et al, 1994). For this reason, these liposomes have also been called 'Stealth' liposomes. Pegylated liposome accumulation in tumours by means of extravasation through leaky vasculature has been demonstrated by electron microscopic examination of C26 colon cancer xenografts and Kaposi's sarcoma-like lesions in mice (Huang et al 1992; 1993). Preclinical studies have shown that cytotoxic drugs entrapped in pegylated liposomes are active against a range of tumours (Mayhew et al, 1992; Vaage et al 1992; 1993*a*, 1993*b*; 1994; 1995; 1999; Williams et al, 1993; Siegal et al, 1995, Newman et al, 1999). In clinical studies, pegylated liposomal doxorubicin has been shown to have substantial activity against AIDS-related Kaposi's sarcoma, (Harrison et al, 1995; Goebel et al, 1996; Northfelt et al, 1998; Stewart et al, 1998) and breast and ovarian cancers (Muggia et al, 1997; Ranson et al, 1997).

In addition, liposomal formulation attenuates the familiar acute (alopecia, nausea and vomiting and local vesicant activity) and chronic (drug-induced cardiomyopathy) toxicities of unencapsulated doxorubicin (Harrison et al, 1995; Madhavan and Northfelt, 1995; Berry et al, 1996). However, alteration of the pharmacokinetics and biodistribution of agents by encapsulation within

*Received 9 December 1997*
*Resubmitted 21 October 1999*
*Accepted 3 December 1999*

*Correspondence to:* KJ Harrington pegylated liposomes has been associated with novel toxic effects. In particular, plantar-palmar erythrodysaesthesia (PPE) or 'hand–foot' syndrome is seen in patients treated with repeated doses of pegylated liposome-encapsulated doxorubicin. This toxicity, which manifests as painful swelling and erythema of the hands, feet, intertriginous areas and sites of trauma, is thought to be the result of accumulation of liposomal doxorubicin in the skin with the effect of delivering a prolonged drug exposure (Gordon et al, 1995).

In view of the flexibility of pegylated liposomes as a delivery system, it is likely that novel therapeutic strategies incorporating a range of anti-cancer agents, including cytotoxic drugs, radiation sensitizers and therapeutic beta-emitting radionuclides, will be investigated. In this study we report in detail the biodistribution and pharmacokinetics of $^{111}$In-DTPA-labelled pegylated liposomes and unencapsulated $^{111}$In-DTPA in nude mice bearing a human head and neck squamous cell cancer xenograft. These data provide important information on the tissues targeted by pegylated liposomes and the extent and time-course of uptake. The potential application of novel targeting approaches are discussed in light of the findings of this study.

### MATERIALS AND METHODS

### Animals and tumour model

Female nude mice of mixed genetic background were used in these experiments. The animals were bred under specific pathogen-free conditions at the Imperial Cancer Research Fund Animal Breeding Unit, South Mimms, Hertfordshire, UK. Thereafter, the animals were housed in sterile filter-top cages on sterile bedding, and maintained on an irradiated diet and autoclaved, acidified water (pH 2.8).

The human tumour KB cell-line was derived from a male patient with a poorly differentiated squamous cell cancer of the floor of mouth and tongue and established in cell culture in 1954 (Eagle, 1955). KB tumour cells were grown to confluence in vitro in RPMI-1640 medium containing 100 U $ml^{-1}$ penicillin and 100 μg $ml^{-1}$ streptomycin, supplemented with 10% foetal calf serum (FCS) (Gibco, Paisley, UK) at 37°C in a humidified atmosphere of 5% $CO_2$ in air. Tumour cells were harvested by brief incubation with trypsin/versene and a single-cell suspension was prepared. Tumour xenografts were established by injecting $5 \times 10^6$ tumour cells in 0.1 ml of culture medium subcutaneously into the right flank of the mice. The animals were used for experiment approximately 14 days after tumour inoculation.

### Liposome labelling with $^{111}$In-oxine

Diethylenetriaminepentaacetic acid (DTPA, Janssen Chimica, Geel, Belgium) was entrapped by SEQUUS™ Pharmaceuticals Inc. in a proprietary pegylated liposome matrix with the following lipid composition (values expressed in % molar ratio): hydrogenated soybean phosphatidylcholine (HSPC) (56.2%), cholesterol (38.3%), and N-(carbamoyl-methoxypolyethylene glycol 2000)-1,2-distearoyl-*sn*-glycero-3-phosphoethanolamine sodium salt (MPEG-DSPE) (5.3%). Liposomes were labelled by incubating 2 ml of $^{111}$In-oxine (Amersham International plc, Amersham, UK) with 20 ml of DTPA-containing pegylated liposomes. Four milligrams of ethylenediaminetetraacetic acid (EDTA) (BDH Ltd, Poole, UK) was added to chelate any residual free $^{111}$In and promote its prompt excretion following intravenous injection. Entrapment of $^{111}$In within the pegylated liposomes was assayed by loading a 10 μl sample on to a 20 ml Sephadex G-50 column (Pharmacia, Uppsala, Sweden). Thirty consecutive 1 ml fractions were eluted with phosphate buffered saline (PBS) and the activity of each fraction was counted in a Canberra Packard Minaxi 5550 gamma counter (Pangbourne, Berkshire, UK). The entrapment was typically found to be greater than 90%.

### Preparation of $^{111}$In-DTPA

40 μl of a solution of $InCl_3$ in 0.04 M HCl containing 22.2 MBq (600 μCi) of radioactivity was titrated to pH 6.0 by the addition of 60 μl of a 3.5% solution of sodium citrate. Thereafter, 10 μl of DTPA, in 10-fold molar excess relative to the $InCl_3$, and 100 μl of a 100 mM solution of sodium acetate (pH 6.0) was added. The final solution was diluted with PBS to a final activity of 10 μCi per 100 μl.

### Determination of biodistribution and pharmacokinetics of $^{111}$In-DTPA-labelled pegylated liposomes

KB tumour-bearing nude mice received 100 μl of $^{111}$In-DTPA-labeled pegylated liposomes, containing 0.37–0.74 MBq (10–20 μCi) of radioactivity, as an intravenous bolus injection via a lateral tail vein. The approximate phospholipid dose per mouse was 1.87 mg of HSPC and 0.64 mg of MPEG-DSPE per 100 μl injection. Mice were killed by exsanguination at 1, 4, 24, 48, 72, 96, 144 and 240 h after the injection of pegylated liposomes. Tumour and normal organs and tissues were dissected at each time-point and their radioactivity was determined by counting pre-weighed tubes in the gamma counter. Standards of the injected material were made in triplicate and used to correct for decay of the $^{111}$In.

### Determination of biodistribution and pharmacokinetics of unencapsulated $^{111}$In-DTPA

KB tumour-bearing nude mice received 100 μl of $^{111}$In-DTPA containing 0.37 MBq (10 μCi) of radioactivity as an intravenous bolus injection via a lateral tail vein. Mice were killed by exsanguination at 5 min, 30 min, 1, 4, 24, 48 and 72 h after the injection of $^{111}$In-DTPA. Tumour and normal organs and tissues were dissected at each time-point and their radioactivity was determined by counting pre-weighed tubes in the gamma counter. Standards of the injected material were made in triplicate and used to correct for decay of the $^{111}$In.

### Incubation of $^{111}InCl_3$, $^{111}$In-DTPA and in vitro stability of $^{111}$In-DTPA-labelled pegylated liposomes in human serum

A solution of $^{111}InCl_3$ containing 3.37 MBq (91.1 μCi) of activity was added to 10 ml of human serum to achieve a final activity of 0.337 MBq $ml^{-1}$ (9.1 μCi $ml^{-1}$), in order to reproduce the maximal initial concentration in mouse serum in the in vivo studies (assuming mouse blood volume to be 1.8 ml and packed cell volume to be 40%). Samples of 500 μl were taken at 5 min, 24 and 120 h, filtered through a 0.2 μm filter (Acrodisc, Gelman Sciences, Ann Arbor, USA) and run on a Superose-6 FPLC column. Eighty fractions of 0.5 mL were collected and counted in the gamma counter.

© 2000 Cancer Research Campaign 

**Table 1** Biodistribution of $^{111}$In-DTPA-labelled pegylated liposomes in nude mice expressed in % injected dose per gram (mean ± SD)

| Tissue | 1 h | 4 h | 24 h | 48 h | 72 h | 96 h | 144 h | 240 h |
|---|---|---|---|---|---|---|---|---|
| Blood | 33.2 (5.4) | 20.6 (1.7) | 7.3 (2.6) | 1.4 (1.0) | 0.2 (0.1) | 0.04 (0.01) | 0.02 (0.00) | 0.01 (0.00) |
| Urine | 56.1 (38.1) | 7.7 (3.5) | 6.0 (2.8) | 9.2 (3.9) | 10.0 (3.3) | 10.0 (9.2) | 4.5 (1.0) | 1.1 (0.4) |
| Tumour | 1.2 (0.3) | 1.8 (0.2) | 5.5 (3.0) | 4.1 (2.0) | 2.7 (0.9) | 1.9 (1.0) | 1.0 (0.4) | 0.4 (0.1) |
| Liver | 6.4 (1.2) | 9.0 (1.2) | 19.3 (2.8) | 17.2 (3.9) | 15.4 (3.7) | 12.8 (3.2) | 9.6 (2.4) | 2.7 (1.0) |
| Spleen | 8.4 (1.7) | 8.5 (0.7) | 18.8 (4.2) | 16.3 (6.4) | 13.4 (4.0) | 12.5 (3.5) | 10.2 (3.0) | 4.7 (0.9) |
| Lung | 7.9 (3.3) | 5.0 (2.6) | 2.2 (0.8) | 1.2 (1.3) | 0.6 (0.2) | 0.4 (0.2) | 0.25 (0.08) | 0.17 (0.04) |
| Kidney | 6.3 (1.6) | 4.8 (0.9) | 6.7 (1.9) | 6.0 (2.7) | 5.3 (2.5) | 4.3 (1.5) | 2.5 (0.6) | 2.2 (0.4) |
| Bladder | 1.2 (0.2) | 1.0 (0.2) | 1.3 (0.3) | 0.6 (0.3) | 0.57 (0.04) | 0.8 (0.3) | 0.38 (0.06) | 0.28 (0.16) |
| Oesophagus | 2.5 (0.5) | 2.4 (1.1) | 4.2 (3.1) | 1.0 (0.4) | 1.0 (0.4) | 1.6 (1.5) | 0.5 (0.1) | 0.2 (0.2) |
| Stomach | 0.8 (0.1) | 0.6 (0.1) | 1.1 (0.4) | 0.9 (0.5) | 0.7 (0.3) | 0.4 (0.1) | 0.3 (0.1) | 0.15 (0.03) |
| Ileum | 1.2 (0.2) | 1.2 (0.2) | 2.0 (0.6) | 1.3 (0.4) | 1.1 (0.3) | 0.8 (0.1) | 0.7 (0.3) | 0.4 (0.2) |
| Colon | 1.2 (0.6) | 0.9 (0.1) | 2.3 (0.6) | 2.2 (0.4) | 1.9 (0.2) | 1.8 (0.7) | 0.5 (0.1) | 0.4 (0.1) |
| Pancreas | 1.1 (0.1) | 1.1 (0.2) | 0.8 (0.4) | 0.30 (0.06) | 0.5 (0.1) | 0.9 (0.9) | 0.25 (0.09) | 0.21 (0.03) |
| Gallbladder | 1.4 (0.8) | 0.5 (0.4) | 0.4 (0.1) | 0.5 (0.4) | 0.09 (0.04) | 0.2 (0.2) | 0.07 (0.05) | 0.10 (0.06) |
| Uterus | 1.6 (0.6) | 2.8 (0.9) | 5.0 (1.3) | 6.6 (2.9) | 4.9 (5.0) | 2.9 (2.3) | 3.4 (1.7) | 2.8 (1.7) |
| Ovary | 1.6 (0.2) | 1.6 (0.4) | 3.5 (0.7) | 2.0 (0.8) | 1.6 (0.6) | 1.6 (0.9) | 1.3 (0.6) | 0.9 (0.4) |
| Skin | 1.1 (0.2) | 1.6 (0.3) | 4.5 (1.4) | 4.2 (1.8) | 4.5 (2.7) | 3.3 (2.3) | 1.6 (0.9) | 1.1 (0.1) |
| Heart | 3.3 (0.4) | 1.8 (0.6) | 1.3 (0.6) | 0.8 (0.6) | 0.8 (0.7) | 0.4 (0.3) | 0.20 (0.10) | 0.21 (0.02) |
| Adrenal | 4.3 (0.7) | 3.4 (0.6) | 2.6 (1.0) | 1.2 (0.3) | 1.2 (0.2) | 0.9 (0.1) | 1.0 (0.2) | 0.4 (0.1) |
| Bone | 1.7 (0.3) | 0.8 (0.1) | 1.1 (0.4) | 0.7 (0.4) | 0.7 (0.4) | 0.6 (0.3) | 0.5 (0.3) | 0.3 (0.1) |
| Muscle | 0.4 (0.1) | 0.31 (0.03) | 0.30 (0.10) | 0.24 (0.13) | 0.16 (0.06) | 0.11 (0.04) | 0.12 (0.08) | 0.08 (0.07) |
| Brain | 0.7 (0.1) | 0.5 (0.1) | 0.13 (0.06) | 0.03 (0.02) | 0.01 (0.00) | 0.01 (0.00) | 0.01 (0.00) | 0.01 (0.00) |
| Spinal cord | 2.1 (0.5) | 1.7 (0.9) | 0.4 (0.3) | 0.09 (0.06) | 0.05 (0.04) | 0.02 (0.00) | 0.03 (0.02) | 0.01 (0.00) |

Similarly, $^{111}$In-DPTA containing 1.69 MBq (45.5 μCi) of activity was added to 5 ml of human serum (0.337 MBq ml$^{-1}$, 9.1 μCi ml$^{-1}$) and incubated at 37°C. Serum samples of 500 μl were taken at 5 min, 4, 24, 48, 72 and 96 h, filtered and run on the FPLC Superose-6 column as described above.

In addition, $^{111}$In-DTPA-labelled pegylated liposomes containing 2.7 MBq (73 μCi) of activity were added to 8 ml of human serum (0.337 MBq ml$^{-1}$, 9.1 μCi ml$^{-1}$) and incubated at 37°C. Serum samples of 500 μl were taken at 30 min, 4, 24, 48, 72, 96 and 240 h, filtered and run on the FPLC Superose-6 column as described above.

### RESULTS

### Pharmacokinetics and biodistribution of $^{111}$In-DTPA-labelled pegylated liposomes

Data were available for a total of 112 animals studied at the various time-points, although not all tissues were sampled for each animal at each time-point. The detailed biodistribution of $^{111}$In-DTPA-labeled pegylated liposomes in nude mice bearing KB head and neck cancer xenograft tumours is presented in Table 1. The mean tumour weight was 0.46 ± 0.39 g (median 0.338, range 0.026–2.083).

The $t_{1/2\alpha}$ and $t_{1/2\beta}$ circulation half-lives of $^{111}$In-DTPA-labeled pegylated liposomes were determined using the P.Fit program (Biosoft, Cambridge, UK) which fitted the blood data for the first 96 h to the biexponential decay equation:

$$y = P\,e^{-\alpha x} + Q\,e^{-\beta x}$$

The $t_{1/2\alpha}$ and $t_{1/2\beta}$ were 1.1 and 10.3 h, respectively. The goodness of fit was within 95% of expected limits for a correct model of the data ($r^2 = 0.996$). The data were also fitted to a monoexponential function which gave a half-life of 10.1 h ($r^2 = 0.972$).

Localization of pegylated liposomes in xenograft tumours was demonstrated at low levels at early time-points, but there was evidence of progressive accumulation to a maximum of 5.5 ± 3.0% ID g$^{-1}$ at 24 h. Thereafter the levels measured in the tumour declined slowly with time (Figure 1). The tumour: blood ratios were 0.04:1 at 1 h, 0.09:1 at 4 h, 0.75:1 at 24 h, 2.9:1 at 48 h, 13.5:1 at 72 h, 47.5:1 at 96 h, 50.0:1 at 144 h and 40.0:1 at 240 h.

The levels of liposome uptake in the clinically relevant tissues of the liver, spleen, heart and skin are shown in Figure 2. Prominent uptake of pegylated liposomes in the liver, spleen and kidneys was demonstrated, reaching maximal levels at 24 h of 19.3 ± 2.8, 18.8 ± 4.2 and 6.7 ± 1.9% ID g$^{-1}$, respectively. Cutaneous deposition of activity increased to a maximum of 4.5 ± 1.4% ID g$^{-1}$ at 24 h and remained at this level until 72 h after injection, whereupon it declined gradually. Significant accumulation of radiolabeled liposomes was seen in the uterus and ovary to maximal levels of 6.6 ± 2.9 and 3.5 ± 0.7% ID g$^{-1}$ at 48 and 24 h, respectively. There was evidence of uptake in tissues of the gastrointestinal tract, although this did not seem to be due to biliary excretion as demonstrated by the low levels of activity in the gallbladder. Liposome uptake in lung, heart, and adrenal glands declined progressively throughout the time course of the study from maximal values at 1 h, with no evidence of progressive accumulation. Very low levels of liposome localization were seen in the tissues of the central nervous system and musculoskeletal system. The high initial levels of $^{111}$In (56.1 ± 38.1% ID g$^{-1}$) measured in the urine at the 1 h time-point almost certainly represent excretion of unencapsulated EDTA-bound radioisotope. The relatively low levels of urinary radioisotope excretion on subsequent days probably reflects the gradual release of $^{111}$In-DTPA from degraded liposomes.

### Pharmacokinetics and biodistribution of unencapsulated $^{111}$In-DTPA

Data were available for a total of 28 mice, four at each dissection time-point. The detailed biodistribution of $^{111}$In-DTPA in nude mice bearing KB head and neck cancer xenograft tumours is

 © 2000 Cancer Research Campaign

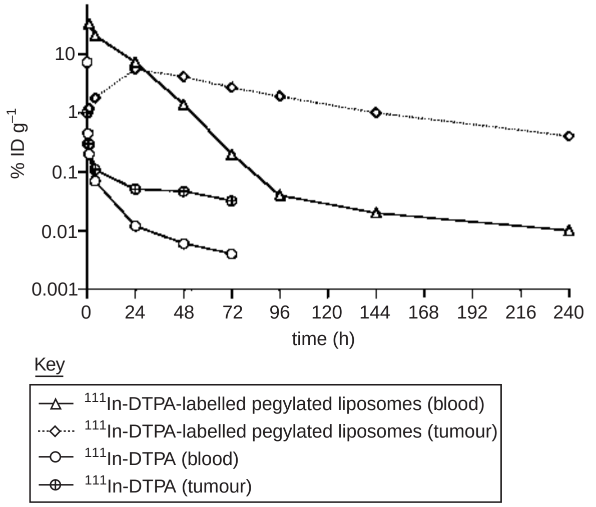

**Figure 1** Blood and tumour levels of $^{111}$In-DTPA-labelled pegylated liposomes and $^{111}$In-DTPA in nude mice bearing KB xenograft tumours.

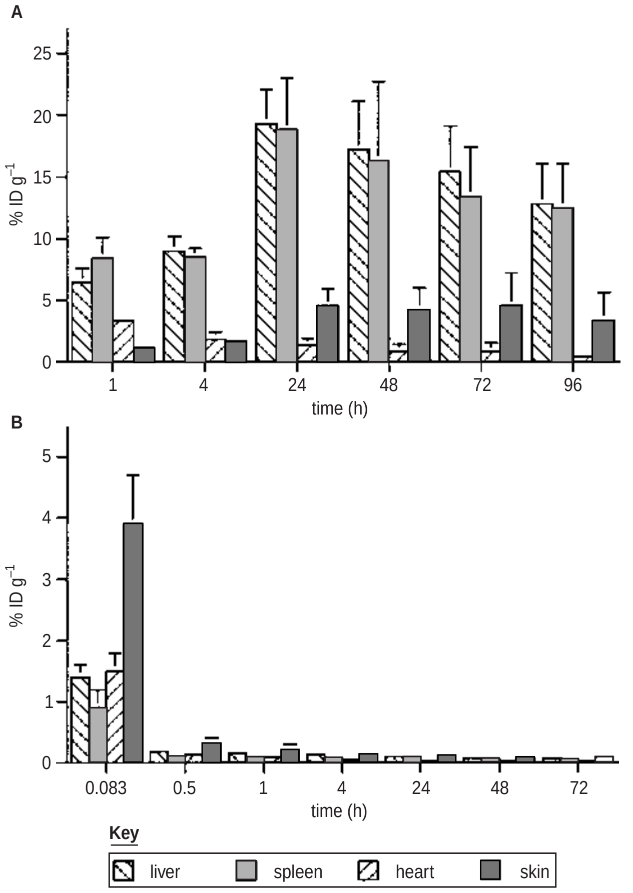

**Figure 2** Biodistribution of (**A**) $^{111}$In-DTPA-labelled pegylated liposomes and (**B**) unencapsulated $^{111}$In-DTPA in nude mice bearing KB xenograft tumours.

presented in Table 2. The mean tumour weight was 0.40 ± 0.26 g (median 0.356, range 0.094–0.912).

The $t_{1/2\alpha}$ and $t_{1/2\beta}$ were 5 min and 1.1 h, respectively. The goodness of fit was within 95% of expected limits for a correct model

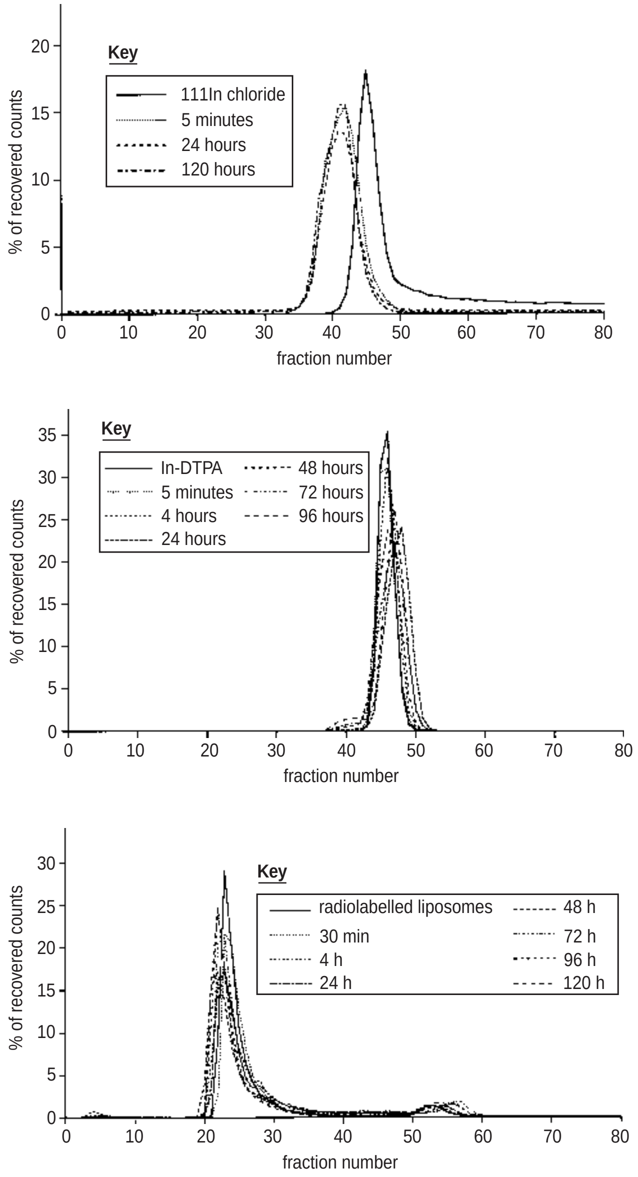

**Figure 3** (**A**) Incubation of $^{111}$In chloride in human serum for 120 h: FPLC Superose-6 column. (**B**) Inclubation of $^{111}$In-DTPA in human serum for 96 h. (**C**) Inclubation of $^{111}$In-DTPA-labelled pegylated liposomes in human serum for 240 h.

of the data. This rapid blood clearance of $^{111}$In-DTPA was associated with very high levels of radioactivity excreted in the urine at the early time-points. Maximum tumour $^{111}$In activity was seen at

5 min. There was no evidence of cumulative deposition of $^{111}$In-DTPA in the tumour or, indeed, in any normal tissue. These data, together with the very short half-life of $^{111}$In-DTPA in the circulation, suggest that $^{111}$In-DTPA in unlikely to become bound to serum proteins to any significant extent.

### Incubation of $^{111}InCl_3$, $^{111}$In-DTPA and in vitro stability of $^{111}$In-DTPA-labeled pegylated liposomes in human serum

The results of running samples of $^{111}InCl_3$ incubated in human serum on the Superose-6 FPLC column are presented in Figure 3A.

© 2000 Cancer Research Campaign

**Table 2** Biodistribution of $^{111}$In-DTPA in nude mice expressed in % injected dose per gram (mean ± SD)

| Tissue | 5 min | 30 min | 1 h | 4 h | 24 h | 48 h | 72 h |
|---|---|---|---|---|---|---|---|
| Blood | 7.2 (1.5) | 0.45 (0.08) | 0.20 (0.03) | 0.07 (0.00) | 0.01 (0.01) | 0.006 (0.000) | 0.004 (0.001) |
| Urine | 783 (956) | 2145 (228) | 756 (117) | 5.1 (3.2) | 0.3 (0.2) | 0.10 (0.01) | 0.07 (0.01) |
| Tumour | 1.0 (0.2) | 0.30 (0.03) | 0.30 (0.03) | 0.11 (0.01) | 0.05 (0.00) | 0.05 (0.02) | 0.03 (0.01) |
| Liver | 1.4 (0.2) | 0.18 (0.04) | 0.15 (0.02) | 0.13 (0.02) | 0.09 (0.01) | 0.07 (0.01) | 0.06 (0.01) |
| Spleen | 0.9 (0.3) | 0.09 (0.01) | 0.08 (0.01) | 0.07 (0.01) | 0.08 (0.01) | 0.06 (0.01) | 0.05 (0.01) |
| Lung | 3.8 (0.8) | 0.32 (0.06) | 0.14 (0.04) | 0.06 (0.01) | 0.04 (0.01) | 0.03 (0.01) | 0.03 (0.01) |
| Kidney | 16.4 (4.8) | 1.6 (0.1) | 1.2 (0.1) | 1.1 (0.2) | 0.8 (0.1) | 0.5 (0.1) | 0.4 (0.2) |
| Bladder | 11.9 (4.9) | 7.0 (3.0) | 4.8 (8.1) | 1.5 (0.6) | 0.23 (0.08) | 0.18 (0.06) | 0.28 (0.13) |
| Oesophagus | 4.3 (0.7) | 0.9 (0.4) | 0.24 (0.15) | 0.20 (0.12) | 0.05 (0.01) | 0.16 (0.25) | 0.13 (0.02) |
| Stomach | 1.3 (0.4) | 0.11 (0.04) | 0.07 (0.02) | 0.16 (0.18) | 0.04 (0.01) | 0.02 (0.01) | 0.02 (0.00) |
| Ileum | 0.9 (0.3) | 0.11 (0.04) | 0.08 (0.03) | 0.12 (0.13) | 0.06 (0.01) | 0.05 (0.02) | 0.03 (0.01) |
| Colon | 1.4 (0.3) | 0.11 (0.03) | 0.07 (0.01) | 0.8 (1.4) | 0.05 (0.01) | 0.03 (0.01) | 0.02 (0.01) |
| Gallbladder | 2.0 (0.9) | 0.3 (0.2) | 0.7 (0.3) | 0.2 (0.1) | 0.03 (0.02) | 0.02 (0.02) | 0.01 (0.01) |
| Pancreas | 1.4 (0.2) | 0.23 (0.02) | 0.06 (0.04) | 0.04 (0.02) | 0.03 (0.01) | 0.03 (0.01) | 0.03 (0.01) |
| Uterus | 8.0 (3.6) | 1.6 (2.3) | 0.2 (0.1) | 0.08 (0.03) | 0.06 (0.03) | 0.07 (0.02) | 0.09 (0.02) |
| Ovary | 4.0 (0.7) | 0.9 (0.3) | 0.1 (0.1) | 0.08 (0.03) | 0.08 (0.03) | 0.05 (0.02) | 0.05 (0.02) |
| Skin | 3.9 (0.8) | 0.3 (0.1) | 0.2 (0.1) | 0.12 (0.01) | 0.10 (0.02) | 0.07 (0.01) | 0.08 (0.02) |
| Heart | 1.5 (0.3) | 0.12 (0.03) | 0.08 (0.01) | 0.04 (0.01) | 0.03 (0.00) | 0.02 (0.01) | 0.02 (0.00) |
| Adrenal | 2.9 (0.4) | 0.27 (0.04) | 1.4 (2.0) | 0.17 (0.03) | 0.09 (0.04) | 0.05 (0.03) | 0.04 (0.03) |
| Bone | 0.7 (0.1) | 0.06 (0.01) | 0.06 (0.03) | 0.04 (0.00) | 0.04 (0.02) | 0.03 (0.01) | 0.02 (0.01) |
| Muscle | 0.8 (0.1) | 0.06 (0.02) | 0.16 (0.24) | 0.03 (0.01) | 0.02 (0.01) | 0.02 (0.01) | 0.01 (0.01) |
| Brain | 0.2 (0.1) | 0.03 (0.00) | 0.16 (0.25) | 0.01 (0.00) | 0.00 (0.00) | 0.00 (0.00) | 0.00 (0.00) |
| Spinal cord | 1.2 (0.7) | 0.08 (0.02) | 0.27 (0.06) | 0.07 (0.03) | 0.01 (0.00) | 0.00 (0.00) | 0.00 (0.00) |

The activity of the $^{111}InCl_3$ control was collected between fractions 39 and 80, with a peak at fraction 45. The sample taken at 5 min showed a significant shift in the distribution of activity to between fractions 33–50, with a peak activity at fraction 42. These results are consistent with rapid binding of the $^{111}$In to serum proteins, most probably transferrin.

The results of running samples of $^{111}$In-DTPA incubated in human serum on the Superose-6 FPLC column are presented in Figure 3B. The activity of the $^{111}$In-DPTA control was collected between fractions 42 and 52, with a peak at fraction 46. The samples taken at the various time-points after addition to serum demonstrated an earlier shoulder to the curve starting at fraction 38. These data suggest that $^{111}$In-DTPA is very stable in serum, with only very low levels of transchelation of the $^{111}$In to serum proteins.

The results of incubation of $^{111}$In-DTPA-labelled pegylated liposomes in human serum over a period of 240 h are presented in Figure 3C. The radioactivity of the radiolabelled pegylated liposomes was collected between fractions 19 and 40 and the free indium activity was collected between fractions 49 and 60 on the FPLC Superose-6 column. As can be seen from Figure 3C, there is very little accumulation of free $^{111}$In-DTPA in the serum and no evidence of significant transchelation of $^{111}$In to a serum protein, which would be expected to run with a peak at about fraction 42.

### DISCUSSION

This study confirms the ability of $^{111}$In-DTPA-labelled pegylated liposomes to target KB xenograft tumours in nude mice and yields a detailed description of their biodistribution in a wide range of normal tissues and organ systems. These data contrast directly with those for the behaviour of unencapsulated $^{111}$In-DTPA, which is cleared very rapidly from the circulation with no evidence of accumulation in either tumour or normal tissues. The absence of leakage of $^{111}$In-DTPA from the pegylated liposomes and the virtual lack of transchelation of $^{111}$In to serum proteins when either $^{111}$In-DTPA-labelled pegylated liposomes or free $^{111}$In-DTPA were incubated in human serum excludes the possibility that the demonstrated uptake is due to targeting by radiolabelled serum elements, such as transferrin.

The data on myocardial and cutaneous uptake agree with the available clinical data for pegylated liposomes containing doxorubicin. Low levels of uptake of $^{111}$In-DTPA-labelled pegylated liposomes were demonstrated in the myocardium, with an area under the time activity curve (AUC) of 132% ID $g^{-1}$ h as compared to 489% ID $g^{-1}$ h for blood for the 240 h period of the study. Importantly, there was no cumulative liposome deposition in cardiac muscle, which correlates well with the documented lack of cardiotoxicity of pegylated liposomal doxorubicin. This contrasts with the high levels of uptake seen in the skin and the profile of cumulative deposition over the first 72 h after injection. The AUC of 615% ID $g^{-1}$ h for the radiolabelled liposomes supports the notion that this would lead to prolonged drug exposure in the skin and provides an explanation of the occurrence of PPE with pegylated liposomal doxorubicin in patients. Therefore, the data generated by this study may provide a useful predictor of the likely adverse effects of pegylated liposomal drug formulations and guide the design and development of novel targeted strategies.

The development of novel liposomal formulations of well established cytotoxic drugs holds the promise of increasing treatment response rates and favorably altering the toxicity profile of the entrapped agents. A number of cytotoxic agents (including anthracyclines, platins and vinca alkaloids) have been successfully packaged within pegylated liposomes and are in various stages of pre-clinical and clinical development (Martin, 1997). In addition, the opportunity to design a range of new liposomally-targeted therapeutic agents may prove to be equally fruitful. For example, preclinical assessment of pegylated liposome-entrapped radiosensitizers and therapeutic beta-emitting radionuclides is proceeding in our laboratory. In the former instance, a number of

 © 2000 Cancer Research Campaign different classes of drugs have been described which have the property of sensitizing tumours to the effects of ionizing radiation (McGinn and Kinsella, 1992; Saunders and Dische, 1996; Britten et al, 1996). Unfortunately, radiosensitization of normal tissues included in the radiation portal and the occurrence of dose-limiting systemic toxicities have severely limited the clinical application of these agents. Liposomal entrapment of radiosensitizers may circumvent these problems by delivering the active agent preferentially to the tumour site, while avoiding deposition in adjacent and distant normal tissues which are sensitive to toxic effects of the unencapsulated agent. From this study, the use of liposomally-targeted radiosensitizers would seem to be a suitable approach for further investigation in patients with cancers of the lung, oesophagus and head and neck. For patients with lung and oesophageal cancers, the dose-limiting late-responding normal structures are the adjacent normal ipsilateral lung tissue, the contralateral lung and the thoracic spinal cord. Radiosensitization in these tissues would be expected to have an adverse effect on outcome and limit the effective radiation dose that could be delivered safely. The low levels of liposome uptake within the lung and thoracic/cervical spinal cord suggest that this would not be a significant risk. Similarly, in patients receiving radical radiotherapy to head and neck cancers, the dose delivered to the cervical spinal cord determines the risk of cervical myelopathy. This study suggests that no significant radiosensitization of this structure would occur with the use of liposomally-entrapped radiosensitizing drugs. Detailed data regarding the localization of liposomes to the normal mucosal structures of the head and neck are not available from this murine model, but an ongoing patient study is presently addressing that issue. The moderate levels of uptake in tissues of the gastrointestinal tract would suggest that liposomally-targeted radiosensitizing drugs would need to be used with caution in patients with pelvic tumours, e.g. cervix, bladder and rectal cancers, although the established radioresponsiveness of these tumour types would certainly justify further examination of this novel approach. On the other hand, the relatively high levels of uptake seen in the liver, spleen and kidneys are likely to preclude the use of liposomal radiosensitizers in treating patients with tumours of the upper abdomen, e.g. pancreatic cancers, because normal tissues might be expected to experience an unacceptable degree of radiosensitization.

The use of liposomally-encapsulated beta-emitting radionuclides as a means of delivering a local radiation boost to the primary tumour and metastatic lymph nodes during a course of external beam radiotherapy may be of clinical use in an analogous fashion to that previously described for radiolabelled monoclonal antibodies (Maraveyas et al, 1995). Clearly this system would be most likely to be of benefit in patients with tumours in regions where liposomal targeting of adjacent critical normal structures would be minimal. For instance, patients with head and neck or lung cancers would be suitable candidates for such an approach, since the normal tissues (liver, spleen and kidneys) receiving a significant radiation dose from radiolabelled liposomes would not receive any of the dose delivered by the external beam radiotherapy. On the other hand, in patients with tumours of the pancreas, the additional liposomally-targeted radiation delivered to the liver, spleen and kidneys would be likely to result in severe toxicity since these tissues would have already received an external beam radiotherapy dose at or near radiotolerance.

### ACKNOWLEDGEMENTS

STEALTH® liposomes are a registered trademark of SEQUUS Pharmaceuticals Inc, Menlo Park, CA, USA.

### REFERENCES

Allen TM and Chonn A (1987) Large unilamellar liposomes with low uptake by the reticuloendothelial system. *FEBS Lett* **223**: 42–46

Bangham AD, Standish HM and Watkins JC (1965) Diffusion of univalent ions across the lamellae of swollen phospholipids. *J Mol Biol* **13**: 238–252

Berry G, Billingham M, Alderman E, Torti F, Lum B, Dumond C and Martin F (1996) Reduced cardiotoxicity of DOXIL (pegylated liposomal doxorubicin) in AIDS Kaposi's sarcoma patients compared to a matched control group of cancer patients given doxorubicin. *Proceedings of the American Society of Clinical Oncologists* **15**: 303 (abstract 843)

Britten RA, Evans AJ, Allalunis-Turner MJ (1996) Effect of cisplatin on the clinically-relevant radiosensitivity of human cervical carcinoma cell lines. *Int J Radiat Oncol Biol Phys* **34**: 367–374

Eagle H. Propagation in a fluid medium of a human epidermoid carcinoma, Strain KB (1955) *Proc Soc Exp Biol Med* **89**: 362–364

Gabizon AA (1994) Liposomal anthracyclines. *Hematol Oncol Clin N America* **8**: 431–450

Gabizon A, Papahadjopoulos D (1988) Liposome formulations with prolonged circulation time in blood and enhanced uptake in tumours. *Proc Natl Acad Sci USA* **85**: 6949–6953

Gabizon A, Catane R, Uziely B, Kaufman B, Safra T, Cohen R, Martin F, Huang A, Barenholz Y (1994) Prolonged circulation time and enhanced accumulation in malignant exudates of doxorubicin encapsulated in polethylene-glycol coated liposomes. *Cancer Res* **54**: 987–992

Goebel F-D, Goldstein D, Goos M, Jablonowski H, Stewart JS (1996) Efficacy and safety of Stealth liposomal doxorubicin in AIDS-related Kaposi's sarcoma. *Br J Cancer* **73**: 989–994

Gordon KB, Tajuddin A, Guitart J, Kuzel TM, Eramo LR, Von Roenn J (1995) Hand-foot syndrome associated with liposome-encapsulated doxorubicin therapy. *Cancer* **75**: 2169–2173

Gregoriades G, Swain CP, Wills EJ, Tavill AS (1974) Drug-carrier potential of liposomes in cancer chemotherapy. *Lancet* **1**: 1313–1316

Harrison M, Tomlinson D, Stewart S (1995) Liposomal entrapped doxorubicin: an active agent in AIDS-related Kaposi's sarcoma. *J Clin Oncol* **13**: 914–920

Huang SK, Lee K-D, Hong K, Friend DS, Papahadjopoulos D (1992) Microscopic localisation of sterically stabilised liposomes in colon carcinoma-bearing mice. *Cancer Res* **52**: 5135–5143

Huang SK, Martin FJ, Jay G, Vogel J, Papahadjopoulos D, Friend DS (1993) Extravasation and transcytosis of liposomes in Kaposi's sarcoma-like dermal lesions of transgenic mice bearing the HIV*tat* gene. *Am J Pathol* **143**: 1–14

Lasic DD, Papahadjopoulos D (1995) Liposomes revisited. *Science* **267**: 1275–1276

Madhavan S, Northfelt DW (1995) Lack of vesicant injury following extravasation of liposomal doxorubicin. *J Natl Cancer Inst* **87**: 1556–1557

Maraveyas A, Stafford N, Rowlinson-Busza G, Stewart JSW, Epenetos AA (1995) Pharmacokinetics, biodistribution and dosimetry of specific and control radiolabelled monoclonal antibodies in patients with primary head and neck squamous cell carcinoma. *Cancer Res* **55**: 1060–1069

Martin FJ (1997) Future prospects for Stealth liposomes in cancer therapy. *Oncology* **11** (suppl 11): 63–68

Mayhew EG, Lasic D, Babbar S, Martin FJ (1992) Pharmacokinetics and antitumour activity of epirubicin encapsulated in long-circulating liposomes incorporating a polyethylene glycol-derivatized phospholipid. *Int J Cancer* **51**: 302–309

McGinn CJ, Kinsella TJ (1992) The experimental and clinical rationale for the use of S-Phase-specific radiosensitisers to overcome tumour cell repopulation. *Semin Oncol* **4**: 21–28

Muggia FM, Hainsworth JD, Jeffers S, Miller P, Groshen S, Tan M, Roman L, Uziely B, Muderspach L, Garcia A, Burnett A, Greco FA, Morrow CP, Paradiso L, Liang L-J (1997) Phase II study of liposomal doxorubicin in refractory ovarian cancer: antitumour activity and toxicity modification by liposomal encapsulation. *J Clin Oncol* **15**: 987–993

Newman MS, Colbern GT, Working PK, Engbers C, Amantea MA (1999) Comparative pharmacokinetics, tissue distribution and therapeutic effectiveness of cisplatin encapsulated in long-circulating, pegylated liposomes (SPI-077) in tumour-bearing mice. *Cancer Chemother Pharmacol* **43**: 1–7

© 2000 Cancer Research Campaign 

Ng TT, Denning DW (1995) Liposomal amphotericin B (AmBisome) therapy in invasive fungal infections. Evaluation of United Kingdom Compassionate use data. *Arch Intern Med* **155**: 1093–1098

Northfelt DW, Dezube B, Thommes JA, Miller BJ, Fischl MA, Friedman-Kien A, Kaplan LD, DuMond C, Mamelok RD, Henry DH (1998) Pegylated-liposomal doxorubicin versus doxorubicin, bleomycin and vincristine in the treatment of AIDS-related Kaposi's sarcoma: results of a randomised phase III clinical trial. *J Clin Oncol* **16**: 2445–2451

Papahadjopoulos D, Allen TM, Gabizon A, Mayhew E, Matthay K, Huang SK, Lee K-D, Woodle MC, Lasic DD, Redemann C, Martin FJ (1991) Sterically stabilised liposomes: Improvements in pharmacokinetics and anti-tumour therapeutic efficacy. *Proc Natl Acad Sci USA* **88**: 11460–11464

Ranson MR, Carmichael J, O'Byrne K, Stewart S, Smith D, Howell A (1997) Treatment of advanced breast cancer with sterically stabilized liposomal doxorubicin: results of a multicentre phase II trial. *J Clin Oncol* **15**: 3185–3191

Russo R, Nigro LC, Minniti S (1996) Visceral leishmaniasis in HIV infected patients: treatment with high dose liposomal amphotericin B (AmBisome). *J Infect* **32**: 133–137

Saunders M, Dische S (1996) Clinical results of hypoxic cell radiosensitisation from hyperbaric oxygen to accelerated radiotherapy, carbogen and nicotinamide. *Br J Cancer* **74**: S271–S278

Siegal T, Horowitz A, Gabizon A (1995) Doxorubicin encapsulated in sterically stabilised liposomes for the treatment of a brain tumour model: biodistribution and therapeutic efficacy. *J Neurosurg* **83**: 1029–1037

Stewart JSW, Jablonowski H, Goebel F-D, Arasteh K, Spittle M, Rios A, Aboulafia D, Galleshaw J, Dezube BJ (1998) Randomised comparative trial of pegylated liposomal doxorubicin versus bleomycin and vincristine in the treatment of AIDS-related Kaposi's sarcoma. International Pegylated Liposomal Doxorubicin Study Group. *J Clin Oncol* **16**: 683–691

Vaage J, Barbera-Guillem E, Abra R, Huang A, Working P (1994) Tissue distribution and therapeutic effect of intravenous free or encapsulated liposomal doxorubicin on human prostate carcinoma xenografts. *Cancer* **73**: 1478–1484

Vaage J, Donovan D, Loftus T, Working P (1995) Prevention of metastasis from mouse mammary carcinomas with liposomes carrying doxorubicin. *Br J Cancer* **72**: 1074–1075

Vaage J, Donovan D, Mayhew E, Uster P, Woodle M (1993*a*) Therapy of mouse mammary carcinomas with vincristine and doxorubicin encapsulated in sterically stabilised liposomes. *Int J Cancer* **54**: 959–964

Vaage J, Mayhew E, Lasic D, Martin F (1992) Therapy of primary and metastatic mouse mammary carcinomas with doxorubicin encapsulated in long circulating liposomes. *Int J Cancer* **51**: 942–948

Vaage J, Donovan D, Mayhew E, Abra R, Huang A (1993*b*) Therapy of human ovarian carcinoma xenografts using doxorubicin encapsulated in sterically stabilised liposomes. *Cancer* **72**: 3671–3675

Vaage J, Donovan D, Wipff E, Abra R, Colbern G, Uster P, Working P (1999) Therapy of a xenografted human colonic carcinoma using cisplatin or doxorubicin encapsulated in long-circulating pegylated stealth liposomes. *Int J Cancer* **80**: 134–137

Williams SS, Alosco TR, Mayhew E, Lasic DD, Martin FJ, Bankert RB (1993) Arrest of human lung tumour xenograft growth in severe combined immunodeficient mice using doxorubicin encapsulated in sterically stabilised liposomes. *Cancer Res* **53**: 3964–3967

 *© 2000 Cancer Research Campaign*
